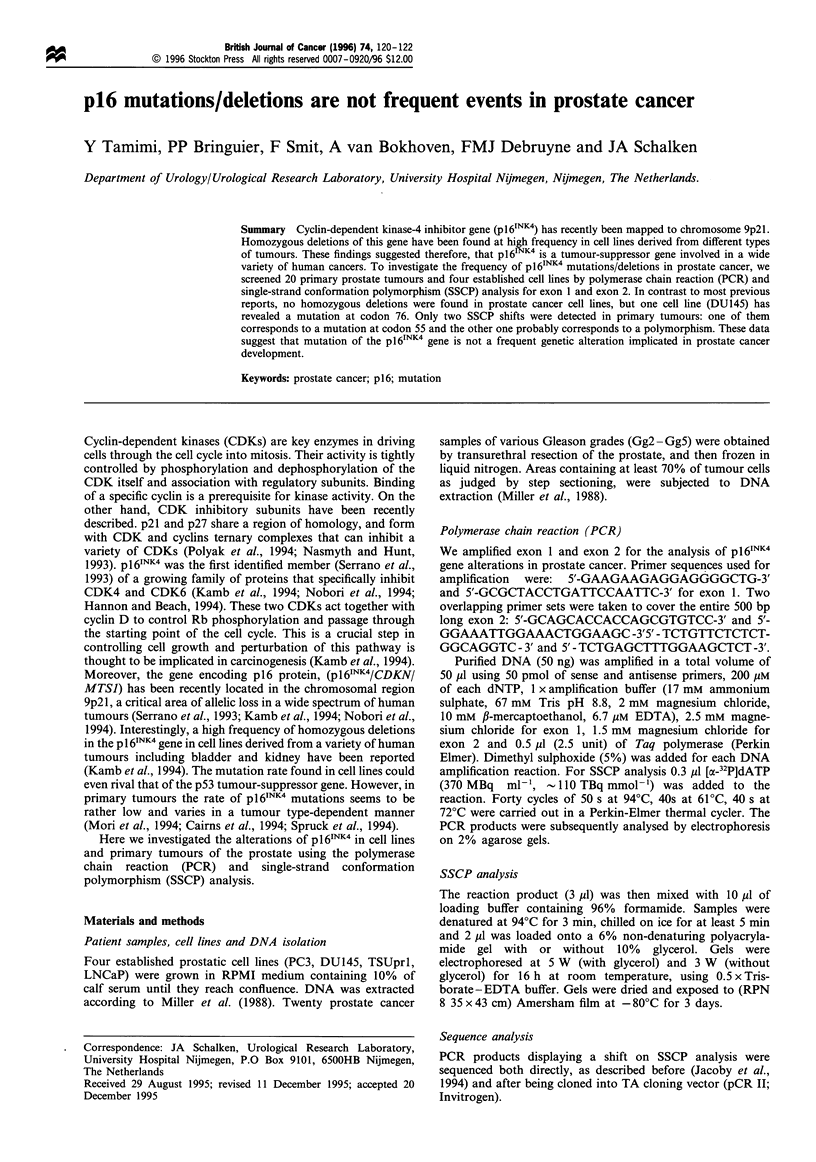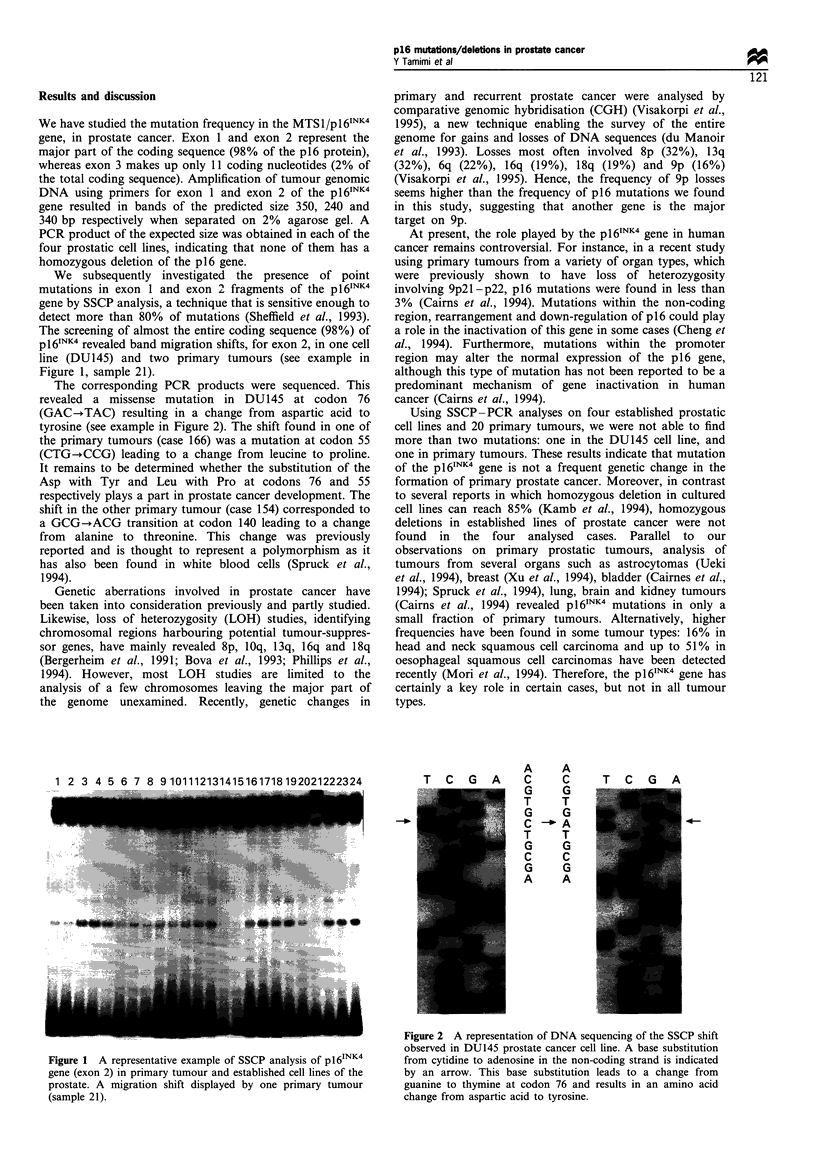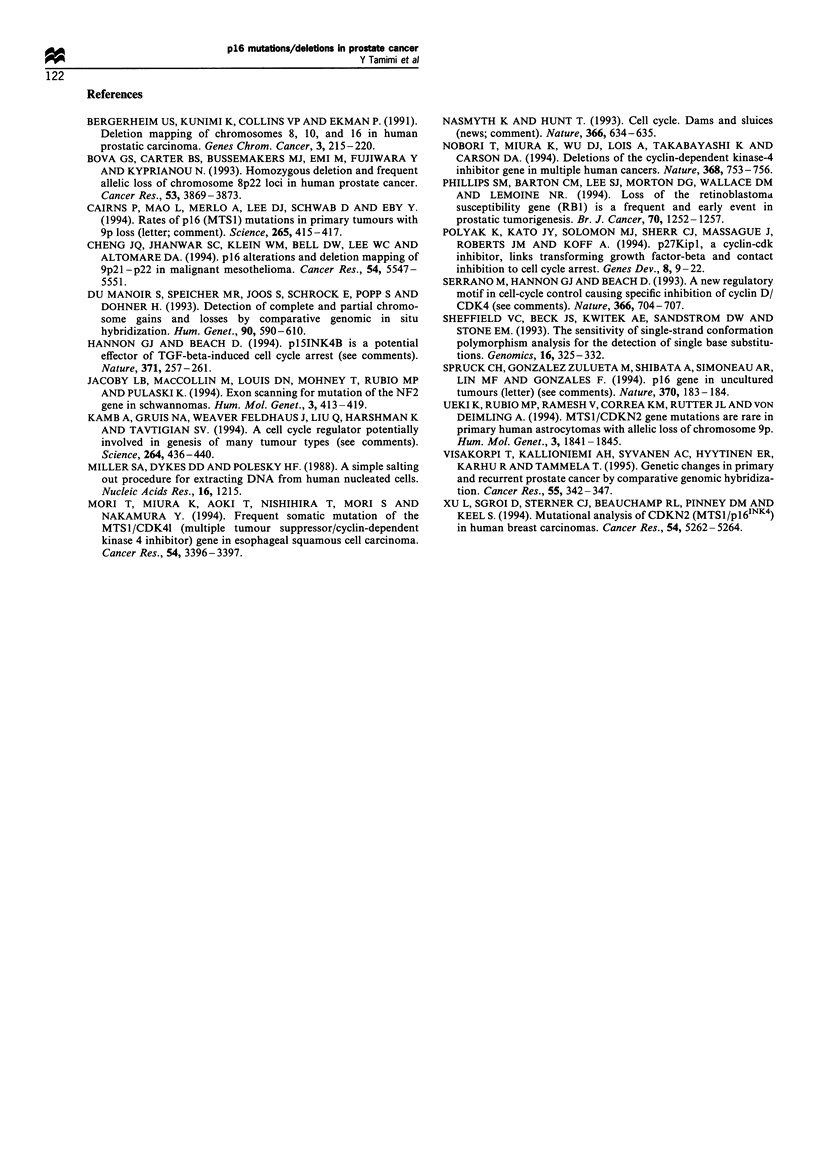# p16 mutations/deletions are not frequent events in prostate cancer.

**DOI:** 10.1038/bjc.1996.325

**Published:** 1996-07

**Authors:** Y. Tamimi, P. P. Bringuier, F. Smit, A. van Bokhoven, F. M. Debruyne, J. A. Schalken

**Affiliations:** Department of Urology/Urological Research Laboratory, University Hospital Nijmegen, The Netherlands.

## Abstract

**Images:**


British Journal of Cancer (1996) 74, 120-122
© 1996 Stockton Press All rights reserved 0007-0920/96 $12.00

### p16 mutations/deletions are not frequent events in prostate cancer

Y Tamimi, PP Bringuier, F Smit, A van Bokhoven, FMJ Debruyne and JA Schalken

*Department of Urology/Urological Research Laboratory, University Hospital Nijmegen, Nijmegen, The Netherlands.*

**Summary** Cyclin-dependent kinase-4 inhibitor gene ($p16^{INK4}$) has recently been mapped to chromosome 9p21. Homozygous deletions of this gene have been found at high frequency in cell lines derived from different types of tumours. These findings suggested therefore, that $p16^{INK4}$ is a tumour-suppressor gene involved in a wide variety of human cancers. To investigate the frequency of $p16^{INK4}$ mutations/deletions in prostate cancer, we screened 20 primary prostate tumours and four established cell lines by polymerase chain reaction (PCR) and single-strand conformation polymorphism (SSCP) analysis for exon 1 and exon 2. In contrast to most previous reports, no homozygous deletions were found in prostate cancer cell lines, but one cell line (DU145) has revealed a mutation at codon 76. Only two SSCP shifts were detected in primary tumours: one of them corresponds to a mutation at codon 55 and the other one probably corresponds to a polymorphism. These data suggest that mutation of the $p16^{INK4}$ gene is not a frequent genetic alteration implicated in prostate cancer development.

**Keywords:** prostate cancer; p16; mutation

Cyclin-dependent kinases (CDKs) are key enzymes in driving cells through the cell cycle into mitosis. Their activity is tightly controlled by phosphorylation and dephosphorylation of the CDK itself and association with regulatory subunits. Binding of a specific cyclin is a prerequisite for kinase activity. On the other hand, CDK inhibitory subunits have been recently described. p21 and p27 share a region of homology, and form with CDK and cyclins ternary complexes that can inhibit a variety of CDKs (Polyak *et al.*, 1994; Nasmyth and Hunt, 1993). $p16^{INK4}$ was the first identified member (Serrano *et al.*, 1993) of a growing family of proteins that specifically inhibit CDK4 and CDK6 (Kamb *et al.*, 1994; Nobori *et al.*, 1994; Hannon and Beach, 1994). These two CDKs act together with cyclin D to control Rb phosphorylation and passage through the starting point of the cell cycle. This is a crucial step in controlling cell growth and perturbation of this pathway is thought to be implicated in carcinogenesis (Kamb *et al.*, 1994). Moreover, the gene encoding p16 protein, ($p16^{INK4}$/*CDKN*/*MTS1*) has been recently located in the chromosomal region 9p21, a critical area of allelic loss in a wide spectrum of human tumours (Serrano *et al.*, 1993; Kamb *et al.*, 1994; Nobori *et al.*, 1994). Interestingly, a high frequency of homozygous deletions in the $p16^{INK4}$ gene in cell lines derived from a variety of human tumours including bladder and kidney have been reported (Kamb *et al.*, 1994). The mutation rate found in cell lines could even rival that of the p53 tumour-suppressor gene. However, in primary tumours the rate of $p16^{INK4}$ mutations seems to be rather low and varies in a tumour type-dependent manner (Mori *et al.*, 1994; Cairns *et al.*, 1994; Spruck *et al.*, 1994).

Here we investigated the alterations of $p16^{INK4}$ in cell lines and primary tumours of the prostate using the polymerase chain reaction (PCR) and single-strand conformation polymorphism (SSCP) analysis.

### Materials and methods

### *Patient samples, cell lines and DNA isolation*

Four established prostatic cell lines (PC3, DU145, TSUprl, LNCaP) were grown in RPMI medium containing 10% of calf serum until they reach confluence. DNA was extracted according to Miller *et al.* (1988). Twenty prostate cancer samples of various Gleason grades (Gg2-Gg5) were obtained by transurethral resection of the prostate, and then frozen in liquid nitrogen. Areas containing at least 70% of tumour cells as judged by step sectioning, were subjected to DNA extraction (Miller *et al.*, 1988).

### *Polymerase chain reaction (PCR)*

We amplified exon 1 and exon 2 for the analysis of $p16^{INK4}$ gene alterations in prostate cancer. Primer sequences used for amplification were: 5′-GAAGAAGAGGAGGGGCTG-3′ and 5′-GCGCTACCTGATTCCAATTC-3′ for exon 1. Two overlapping primer sets were taken to cover the entire 500 bp long exon 2: 5′-GCAGCACCACCAGCGTGTCC-3′ and 5′-GGAAATTGGAAACTGGAAGC-3′5′-TCTGTTCTCTCT-GGCAGGTC-3′ and 5′-TCTGAGCTTTGGAAGCTCT-3′.

Purified DNA (50 ng) was amplified in a total volume of 50 μl using 50 pmol of sense and antisense primers, 200 μM of each dNTP, 1 × amplification buffer (17 mM ammonium sulphate, 67 mM Tris pH 8.8, 2 mM magnesium chloride, 10 mM β-mercaptoethanol, 6.7 μM EDTA), 2.5 mM magnesium chloride for exon 1, 1.5 mM magnesium chloride for exon 2 and 0.5 μl (2.5 unit) of *Taq* polymerase (Perkin Elmer). Dimethyl sulphoxide (5%) was added for each DNA amplification reaction. For SSCP analysis 0.3 μl [α-$^{32}$P]dATP (370 MBq $ml^{-1}$, ~110 TBq $mmol^{-1}$) was added to the reaction. Forty cycles of 50 s at 94°C, 40s at 61°C, 40 s at 72°C were carried out in a Perkin-Elmer thermal cycler. The PCR products were subsequently analysed by electrophoresis on 2% agarose gels.

### *SSCP analysis*

The reaction product (3 μl) was then mixed with 10 μl of loading buffer containing 96% formamide. Samples were denatured at 94°C for 3 min, chilled on ice for at least 5 min and 2 μl was loaded onto a 6% non-denaturing polyacrylamide gel with or without 10% glycerol. Gels were electrophoresed at 5 W (with glycerol) and 3 W (without glycerol) for 16 h at room temperature, using 0.5 × Tris-borate-EDTA buffer. Gels were dried and exposed to (RPN 8 35 × 43 cm) Amersham film at −80°C for 3 days.

### *Sequence analysis*

PCR products displaying a shift on SSCP analysis were sequenced both directly, as described before (Jacoby *et al.*, 1994) and after being cloned into TA cloning vector (pCR II; Invitrogen).

Correspondence: JA Schalken, Urological Research Laboratory, University Hospital Nijmegen, P.O Box 9101, 6500HB Nijmegen, The Netherlands
Received 29 August 1995; revised 11 December 1995; accepted 20 December 1995

### Results and discussion

We have studied the mutation frequency in the MTS1/p16$^{INK4}$ gene, in prostate cancer. Exon 1 and exon 2 represent the major part of the coding sequence (98% of the p16 protein), whereas exon 3 makes up only 11 coding nucleotides (2% of the total coding sequence). Amplification of tumour genomic DNA using primers for exon 1 and exon 2 of the p16$^{INK4}$ gene resulted in bands of the predicted size 350, 240 and 340 bp respectively when separated on 2% agarose gel. A PCR product of the expected size was obtained in each of the four prostatic cell lines, indicating that none of them has a homozygous deletion of the p16 gene.

We subsequently investigated the presence of point mutations in exon 1 and exon 2 fragments of the p16$^{INK4}$ gene by SSCP analysis, a technique that is sensitive enough to detect more than 80% of mutations (Sheffield *et al*., 1993). The screening of almost the entire coding sequence (98%) of p16$^{INK4}$ revealed band migration shifts, for exon 2, in one cell line (DU145) and two primary tumours (see example in Figure 1, sample 21).

The corresponding PCR products were sequenced. This revealed a missense mutation in DU145 at codon 76 (GAC→TAC) resulting in a change from aspartic acid to tyrosine (see example in Figure 2). The shift found in one of the primary tumours (case 166) was a mutation at codon 55 (CTG→CCG) leading to a change from leucine to proline. It remains to be determined whether the substitution of the Asp with Tyr and Leu with Pro at codons 76 and 55 respectively plays a part in prostate cancer development. The shift in the other primary tumour (case 154) corresponded to a GCG→ACG transition at codon 140 leading to a change from alanine to threonine. This change was previously reported and is thought to represent a polymorphism as it has also been found in white blood cells (Spruck *et al*., 1994).

Genetic aberrations involved in prostate cancer have been taken into consideration previously and partly studied. Likewise, loss of heterozygosity (LOH) studies, identifying chromosomal regions harbouring potential tumour-suppressor genes, have mainly revealed 8p, 10q, 13q, 16q and 18q (Bergerheim *et al*., 1991; Bova *et al*., 1993; Phillips *et al*., 1994). However, most LOH studies are limited to the analysis of a few chromosomes leaving the major part of the genome unexamined. Recently, genetic changes in primary and recurrent prostate cancer were analysed by comparative genomic hybridisation (CGH) (Visakorpi *et al*., 1995), a new technique enabling the survey of the entire genome for gains and losses of DNA sequences (du Manoir *et al*., 1993). Losses most often involved 8p (32%), 13q (32%), 6q (22%), 16q (19%), 18q (19%) and 9p (16%) (Visakorpi *et al*., 1995). Hence, the frequency of 9p losses seems higher than the frequency of p16 mutations we found in this study, suggesting that another gene is the major target on 9p.

At present, the role played by the p16$^{INK4}$ gene in human cancer remains controversial. For instance, in a recent study using primary tumours from a variety of organ types, which were previously shown to have loss of heterozygosity involving 9p21–p22, p16 mutations were found in less than 3% (Cairns *et al*., 1994). Mutations within the non-coding region, rearrangement and down-regulation of p16 could play a role in the inactivation of this gene in some cases (Cheng *et al*., 1994). Furthermore, mutations within the promoter region may alter the normal expression of the p16 gene, although this type of mutation has not been reported to be a predominant mechanism of gene inactivation in human cancer (Cairns *et al*., 1994).

Using SSCP–PCR analyses on four established prostatic cell lines and 20 primary tumours, we were not able to find more than two mutations: one in the DU145 cell line, and one in primary tumours. These results indicate that mutation of the p16$^{INK4}$ gene is not a frequent genetic change in the formation of primary prostate cancer. Moreover, in contrast to several reports in which homozygous deletion in cultured cell lines can reach 85% (Kamb *et al*., 1994), homozygous deletions in established lines of prostate cancer were not found in the four analysed cases. Parallel to our observations on primary prostatic tumours, analysis of tumours from several organs such as astrocytomas (Ueki *et al*., 1994), breast (Xu *et al*., 1994), bladder (Cairnes *et al*., 1994); Spruck *et al*., 1994), lung, brain and kidney tumours (Cairns *et al*., 1994) revealed p16$^{INK4}$ mutations in only a small fraction of primary tumours. Alternatively, higher frequencies have been found in some tumour types: 16% in head and neck squamous cell carcinoma and up to 51% in oesophageal squamous cell carcinomas have been detected recently (Mori *et al*., 1994). Therefore, the p16$^{INK4}$ gene has certainly a key role in certain cases, but not in all tumour types.

**Figure 1** A representative example of SSCP analysis of p16$^{INK4}$ gene (exon 2) in primary tumour and established cell lines of the prostate. A migration shift displayed by one primary tumour (sample 21).

**Figure 2** A representation of DNA sequencing of the SSCP shift observed in DU145 prostate cancer cell line. A base substitution from cytidine to adenosine in the non-coding strand is indicated by an arrow. This base substitution leads to a change from guanine to thymine at codon 76 and results in an amino acid change from aspartic acid to tyrosine.

### References

BERGERHEIM US, KUNIMI K, COLLINS VP AND EKMAN P. (1991). Deletion mapping of chromosomes 8, 10, and 16 in human prostatic carcinoma. *Genes Chrom. Cancer*, **3**, 215–220.

BOVA GS, CARTER BS, BUSSEMAKERS MJ, EMI M, FUJIWARA Y AND KYPRIANOU N. (1993). Homozygous deletion and frequent allelic loss of chromosome 8p22 loci in human prostate cancer. *Cancer Res.*, **53**, 3869–3873.

CAIRNS P, MAO L, MERLO A, LEE DJ, SCHWAB D AND EBY Y. (1994). Rates of p16 (MTS1) mutations in primary tumours with 9p loss (letter; comment). *Science*, **265**, 415–417.

CHENG JQ, JHANWAR SC, KLEIN WM, BELL DW, LEE WC AND ALTOMARE DA. (1994). p16 alterations and deletion mapping of 9p21–p22 in malignant mesothelioma. *Cancer Res.*, **54**, 5547–5551.

DU MANOIR S, SPEICHER MR, JOOS S, SCHROCK E, POPP S AND DOHNER H. (1993). Detection of complete and partial chromosome gains and losses by comparative genomic in situ hybridization. *Hum. Genet.*, **90**, 590–610.

HANNON GJ AND BEACH D. (1994). p15INK4B is a potential effector of TGF-beta-induced cell cycle arrest (see comments). *Nature*, **371**, 257–261.

JACOBY LB, MACCOLLIN M, LOUIS DN, MOHNEY T, RUBIO MP AND PULASKI K. (1994). Exon scanning for mutation of the NF2 gene in schwannomas. *Hum. Mol. Genet.*, **3**, 413–419.

KAMB A, GRUIS NA, WEAVER FELDHAUS J, LIU Q, HARSHMAN K AND TAVTIGIAN SV. (1994). A cell cycle regulator potentially involved in genesis of many tumour types (see comments). *Science*, **264**, 436–440.

MILLER SA, DYKES DD AND POLESKY HF. (1988). A simple salting out procedure for extracting DNA from human nucleated cells. *Nucleic Acids Res.*, **16**, 1215.

MORI T, MIURA K, AOKI T, NISHIHIRA T, MORI S AND NAKAMURA Y. (1994). Frequent somatic mutation of the MTS1/CDK4I (multiple tumour suppressor/cyclin-dependent kinase 4 inhibitor) gene in esophageal squamous cell carcinoma. *Cancer Res.*, **54**, 3396–3397.

NASMYTH K AND HUNT T. (1993). Cell cycle. Dams and sluices (news; comment). *Nature*, **366**, 634–635.

NOBORI T, MIURA K, WU DJ, LOIS A, TAKABAYASHI K AND CARSON DA. (1994). Deletions of the cyclin-dependent kinase-4 inhibitor gene in multiple human cancers. *Nature*, **368**, 753–756.

PHILLIPS SM, BARTON CM, LEE SJ, MORTON DG, WALLACE DM AND LEMOINE NR. (1994). Loss of the retinoblastoma susceptibility gene (RB1) is a frequent and early event in prostatic tumorigenesis. *Br. J. Cancer*, **70**, 1252–1257.

POLYAK K, KATO JY, SOLOMON MJ, SHERR CJ, MASSAGUE J, ROBERTS JM AND KOFF A. (1994). p27Kip1, a cyclin-cdk inhibitor, links transforming growth factor-beta and contact inhibition to cell cycle arrest. *Genes Dev.*, **8**, 9–22.

SERRANO M, HANNON GJ AND BEACH D. (1993). A new regulatory motif in cell-cycle control causing specific inhibition of cyclin D/CDK4 (see comments). *Nature*, **366**, 704–707.

SHEFFIELD VC, BECK JS, KWITEK AE, SANDSTROM DW AND STONE EM. (1993). The sensitivity of single-strand conformation polymorphism analysis for the detection of single base substitutions. *Genomics*, **16**, 325–332.

SPRUCK CH, GONZALEZ ZULUETA M, SHIBATA A, SIMONEAU AR, LIN MF AND GONZALES F. (1994). p16 gene in uncultured tumours (letter) (see comments). *Nature*, **370**, 183–184.

UEKI K, RUBIO MP, RAMESH V, CORREA KM, RUTTER JL AND VON DEIMLING A. (1994). MTS1/CDKN2 gene mutations are rare in primary human astrocytomas with allelic loss of chromosome 9p. *Hum. Mol. Genet.*, **3**, 1841–1845.

VISAKORPI T, KALLIONIEMI AH, SYVANEN AC, HYYTINEN ER, KARHU R AND TAMMELA T. (1995). Genetic changes in primary and recurrent prostate cancer by comparative genomic hybridization. *Cancer Res.*, **55**, 342–347.

XU L, SGROI D, STERNER CJ, BEAUCHAMP RL, PINNEY DM AND KEEL S. (1994). Mutational analysis of CDKN2 (MTS1/p16$^{INK4}$) in human breast carcinomas. *Cancer Res.*, **54**, 5262–5264.